# Discovery of fibrillar adhesins across bacterial species

**DOI:** 10.1186/s12864-021-07586-2

**Published:** 2021-07-18

**Authors:** Vivian Monzon, Aleix Lafita, Alex Bateman

**Affiliations:** European Molecular Biology Laboratory, European Bioinformatics Institute (EMBL-EBI), Wellcome Genome Campus, Hinxton, CB10 1SD UK

**Keywords:** Bacterial surface proteins, Adhesive protein domain, Host-pathogen interaction, Sequence analysis, Pfam, Protein domain architecture, Multidomain protein

## Abstract

**Background:**

Fibrillar adhesins are long multidomain proteins that form filamentous structures at the cell surface of bacteria. They are an important yet understudied class of proteins composed of adhesive and stalk domains that mediate interactions of bacteria with their environment. This study aims to characterize fibrillar adhesins in a wide range of bacterial phyla and to identify new fibrillar adhesin-like proteins to improve our understanding of host-bacteria interactions.

**Results:**

Through careful literature and computational searches, we identified 82 stalk and 27 adhesive domain families in fibrillar adhesins. Based on the presence of these domains in the UniProt Reference Proteomes database, we identified and analysed 3,542 fibrillar adhesin-like proteins across species of the most common bacterial phyla. We further enumerate the adhesive and stalk domain combinations found in nature and demonstrate that fibrillar adhesins have complex and variable domain architectures, which differ across species. By analysing the domain architecture of fibrillar adhesins, we show that in Gram positive bacteria, adhesive domains are mostly positioned at the N-terminus and cell surface anchors at the C-terminus of the protein, while their positions are more variable in Gram negative bacteria. We provide an open repository of fibrillar adhesin-like proteins and domains to enable further studies of this class of bacterial surface proteins.

**Conclusion:**

This study provides a domain-based characterization of fibrillar adhesins and demonstrates that they are widely found in species across the main bacterial phyla. We have discovered numerous novel fibrillar adhesins and improved our understanding of pathogenic adhesion and invasion mechanisms.

**Supplementary Information:**

The online version contains supplementary material available at (10.1186/s12864-021-07586-2).

## Background

Studying how bacteria interact with their host is essential for understanding bacterial infection processes and characterizing interactions with commensal bacteria. Adhesion to the host cell is the first step of a bacterial infection. Interfering in the adhesion process by Anti-adhesion therapies, primarily by stimulating a humoral immune response, is an effective strategy to prevent infections and has been widely used for the development of vaccines targeting host binding proteins [1]. For example, advanced vaccine candidates target the binding domain of the M protein of Group A Streptococcus bacteria [2].

**Fig. 1:**
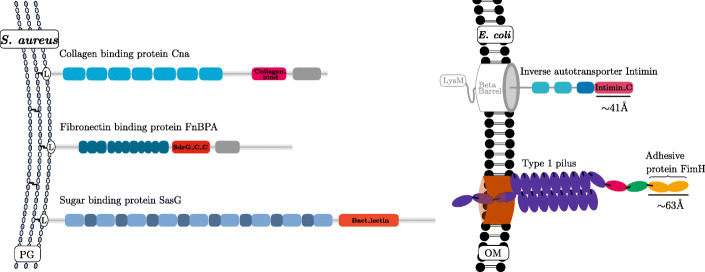
Schematic representation of fibrillar adhesins domain architecture and comparison to a pilus polymer: Three well studied fibrillar adhesins from *S. aureus* are shown on the left. The stalk domains (blue), adhesive domains (red) and host ligand targets (not shown) differ between these proteins. All three proteins are anchored to the peptidoglycan (PG) layer by the gram positive sortase anchor motif LPxTG, here indicated with the L. On the right, a direct comparison of the Intimin fibrillar adhesin (adapted from [7]) compared to the Type 1 pilus (adapted from [8]) in *E. coli* is shown. Compared to the monomeric inverse autotransporter Intimin, the Type 1 pilus is polymeric with the FimH adhesive protein expressed at its tip. The fibrillar adhesin Intimin is anchored by its beta barrel domain in the outer membrane (OM)

Fibrillar adhesins are long filamentous surface proteins that share similar functions and are expressed by a variety of different bacteria [3]. Besides mediating interactions between bacteria and host cells, fibrillar adhesins can be involved in biofilm formation and other cell-cell interactions [4, 5]. The name “fibrillar adhesin” is based on their characteristic filamentous appearance when viewed by electron microscopy. Compared to a second type of large filamentous surface adhesin, namely pili or fimbriae, fibrillar adhesins are composed out of large arrays of domains in a single protein chain, often repeated in tandem, instead of multiple protein subunits. These domains are named stalk domains for their function as elongated rod-like structures, which project the functional part of the protein, the adhesive domain, towards the target host cell [6] (Fig. 1). Compared to pili or fimbriae, fibrillar adhesins are thinner, more flexible [9] and more than 10 times smaller than type 1 pili, which are typically between 0.3 and 2.5 micrometers long [10].

Few fibrillar adhesins have been studied so far and these come from a limited range of organisms. To our knowledge, no comprehensive review or study of fibrillar adhesins across bacterial species has been performed before. It would be infeasible to experimentally characterise these proteins at large scale and determine their fibrillar nature. In this work, we aim to computationally identify fibrillar adhesin proteins using a sequence-based perspective. Thus, we define a new class of proteins that we call fibrillar adhesin-like (FA-like) proteins based on the common features of known fibrillar adhesins. These proteins have the following properties:

- FA-like proteins are extracellular.
- FA-like proteins possess an adhesive region usually at the protein terminus.
- FA-like proteins possess a stalk-like region that forms a rigid rod-like structure.
- FA-like proteins usually possess a surface anchoring motif or domain.
- FA-like proteins are composed of one or a small number of identical fibrillar chains, where each monomer extends through the whole length of the fibril.

Fibrillar adhesins appear to be composed of a relatively limited number of domain families, which facilitates their computational identification. We search for FA-like proteins based on a well studied collection of adhesive and stalk domains and we use Pfam domain definitions to identify and characterize FA-like proteins. This study aims to create a comprehensive survey of fibrillar adhesins across bacterial phyla to gain understanding of their prevalence, distribution and composition. We hope that this study will guide researchers to investigate interesting new fibrillar adhesins, leading to an improvement in our understanding of microbial interactions in health and disease.

## Results

### Detection of FA-like proteins

Fibrillar adhesins are composed of multiple stalk domains, commonly repeated in tandem, and at least one adhesive domain. We gathered a set of 27 adhesive Pfam domain families from known fibrillar adhesins in the literature and 82 stalk domain families from proteins with tandem sequence repeats, as described in the “Methods” section. These 82 stalk domains include 6 new domain families, which were built in the course of this study (Pfam: PF19403 - PF19408). These domain families were used to identify FA-like proteins.

The majority (19 out of 27) of the adhesive domains in our set bind to protein ligands, while 7 out of 27 bind to carbohydrates (supplementary table S2). The Ice_binding domain (Pfam: PF11999) differs from the other adhesive domains in that it doesn’t bind to host cells or to other bacteria, but to ice crystals, allowing organisms to survive in extremely cold environments [11].

**Fig. 2:**
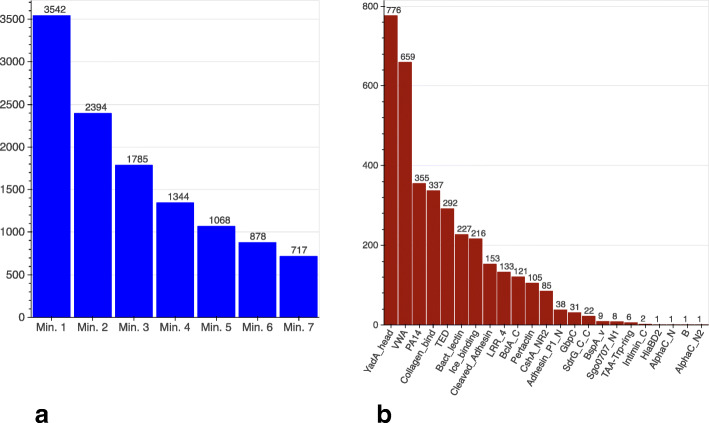
Minimum number of stalk domains per protein and proportion of identified adhesive domains in FA-like proteins: **a** Number of FA-like proteins identified depending on the number of required stalk domains. All annotated stalk domains are counted, regardless whether or not they belong to the same or different stalk domain families. **b** Frequency of adhesive domains in combination with at least one known stalk domain. For proteins with multiple adhesive domains, each domain family is counted once per protein. Throughout this study, we will use blue colouring for stalk domains and red colouring for adhesive domains

We identified a total of 3,542 FA-like proteins composed of at least one adhesive and at least one stalk domain from our sets. These represent 0.013% of all proteins in the UniProt bacterial reference proteomes. Species-wise, FA-like proteins are found in 26% of the bacterial reference proteomes [12]. The large majority of these proteins are unstudied and therefore interesting targets for further investigations. Only 9% of the FA-like proteins are annotated with the Gene Ontology (GO) term cell adhesion in the UniProt database [12]. Some of these newly identified proteins are found in well studied bacteria, for example we predicted three potential new internalin proteins (UniProt: Q8Y7I8, Q8Y8U2, Q8Y9W0) in *Listeria monocytogenes*. Different combinations of adhesive and stalk domains are found across FA-like proteins. The total number of stalk domains in FA-like proteins varies from 1 to 73. Figure 2a shows the number of FA-like proteins identified for different numbers of stalk domains, regardless whether they belong to the same or to a different Pfam family. The largest number of FA-like proteins are found when only one stalk domain is required in addition to the adhesive domain; our study is based on that set.

Despite some of the FA-like proteins identified having a low number of annotated stalk domains, the actual number might be higher, since some stalk domains are not yet known and therefore not annotated. In addition, the expression of FA-like proteins with a variety of stalk domain numbers leads to length variation, which is suggested to play a role in the regulation of the bacterial interactions with its binding partner [13]. Nevertheless, in 1,785 FA-like proteins three or more stalk domains were found in combination with an adhesive domain.

### Prevalence of adhesive domains

Bacteria use a wide range of different protein domains to mediate adherence to their host or other organisms. To date, it is not known which of these domains are the most common within fibrillar adhesins. Our domain based screen for FA-like proteins (Fig. 2b) shows that the most commonly found adhesive domain in combination with at least one known stalk domain is YadA_head (Pfam: PF05658), found in 776 FA-like proteins (22%), followed by VWA (Pfam: PF00092) with 659 hits (19%) and PA14 (Pfam: PF07691) found in 355 FA-like proteins (10%).

### Domain combinations of FA-like proteins

We were further interested to investigate if there is a common domain grammar in FA-like proteins. In the detected FA-like proteins, we found combinations of 76 different stalk domains with 23 different adhesive domains. SlpA (Pfam: PF03217), I-set (Pfam: PF07679) Fil_haemagg (Pfam: PF05594), Fil_haemagg_2 (Pfam: PF13332), DUF5649 (Pfam: PF18886) and Beta_helix_3 (Pfam: PF18889) were the only stalk domains not found in combination with a known adhesive domain and the adhesive domains SabA_adhesion (Pfam: PF18304), SSURE (Pfam: PF11966), FadA (Pfam: PF09403) and FimH_man-bind (Pfam: PF09160) were not found with any known stalk domains.

**Fig. 3:**
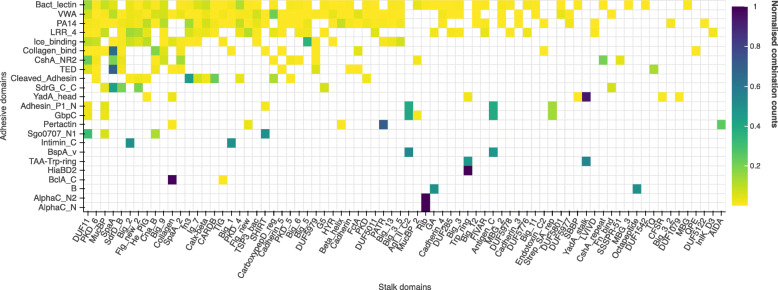
Stalk and adhesive domain combinations in FA-like proteins from the UniProt reference proteomes: The x-axis of this heatmap represents the known stalk domains and the y-axis the known adhesive domains. Multiple domains from the same domain family found in the same protein were only counted once

We plotted the combinations of adhesive and stalk domains as a heatmap in Fig. 3. Some adhesive domains are promiscuous and found in combination with a wide range of different stalk domains, like the PA14 domain (Pfam: PF07691), whereas others are only found in combination with a single stalk domain, such as the AlphaC_N domain (Pfam: PF08829), which only appears in combination with Rib domains. Certain domains are mostly found together in a specific protein type, for example Adhesin_P1_N (Pfam: PF18652) and GbpC (Pfam: PF08363) in combination with the stalk domains Antigen_C (Pfam: PF16364), AgI_II_C2 (Pfam: PF17998) and Strep_SA_rep (Pfam: PF06696), found in the *Streptococcus mutans* adhesin P1 protein [14].

The pair YadA_head and YadA_stalk (Pfam: PF05662) are the most prevalent domain combination, found in 770 FA-like proteins. YadA_stalk is by far the most commonly occurring stalk domain with annotations in 772 of the 3,542 FA-like proteins, followed by SpaA (Pfam: PF17802), with 668 FA-like protein hits (supplementary figure S1). Collagen_bind (Pfam: PF05737) and SpaA are the second most prevalent domain combination found in 303 FA-like proteins.

There are likely more adhesive and stalk domains not yet known and therefore not included in this study. Furthermore, we only used proteins from the UniProt Reference Proteomes, so potential FA-like proteins and novel domain combinations might be missing in this study. Moreover, imperfect annotation of stalk or adhesive domains by the Pfam hidden Markov models (HMMs) may lead to further missing FA-like proteins. One example is the SAP077A_019 fibrillar adhesin of *S. aureus* (UniProt: D2JAN8) with AlphaC_N and AlphaC_N2 adhesive domains in combination with Big_6 stalk domains. SAP077A_019 is not part of the UniProt Reference Proteomes and its adhesive domains are not captured by the current Pfam models.

Besides combinations of adhesive and stalk domains, which occur together in the same protein, there are also stalk domains that are commonly found together (supplementary figure S2). SpaA and Cna_B (Pfam: PF05738) are the stalk domains most often found together, in a total of 99 FA-like proteins. Both domain families belong to the Transthyretin clan (Pfam: CL0287), indicating that they are homologous and possess the same fold. SpaA is found together with a wide range of stalk domains, several of which belong to the Transthyretin clan and others belong to different clans such as the Big_9 domain of the E-set clan (Pfam: CL0159). Out of the 76 stalk domains which are found in combination with an adhesive domain, there are six domains which are not found with another known stalk domain on the same protein (in the Reference Proteomes). These five domains are Rib (Pfam: PF08428), QPE (Pfam: PF18874), Big_3 (Pfam: PF07523), Big_3_2 (Pfam: PF12245), DUF5977 (Pfam: PF19404) and AIDA (Pfam: PF16168).

Domain pairs that are annotated together in the same protein and that function together are called supra-domains [15]. In 222 out of the 337 FA-like proteins with Collagen_bind and in 20 of 22 FA-like proteins with SdrG_C_C (Pfam: PF10425), a Big_8 (Pfam: PF17961) domain was found positioned N-terminally within 50 amino acid distance to the SdrG_C_C or Collagen_bind domain. The principles of the binding mechanisms of SdrG_C_C, called Dock lock latch (DLL) mechanism and of Collagen_bind, called Collagen-hug, are comparable. Both, in the DLL as well as in the Collagen-hug binding mechanism, two domains build a trench in which the ligand docks and is finally locked. By comparing the sequence annotation with the structure of the respective binding mechanism, it is clear that the Big_8 is essential for the binding mechanism. Thus we define two supradomains as Big_8:SdrG_C_C and Big_8:Collagen_bind. Aligning the structures of both adhesive domains showed that they are structurally similar and likely to be homologous with a RMSD < 5Å’ [16].

Another example of a supra-domain, present on the heatmap in Fig. 3, is the AlphaC_N and AlphaC_N2 domain, which form the N-terminal domain in combination with the Rib stalk domain repeats in the Alpha C protein in Group B Streptococcus [17].

### FA-like protein cell anchoring

Fibrillar adhesins are surface proteins that are attached to the bacterial cell surface. The attachment mechanism can take many forms, such as non-covalent attachment by S-layer homology domains [18], or covalent anchoring via a sortase anchoring motif.

**Fig. 4:**
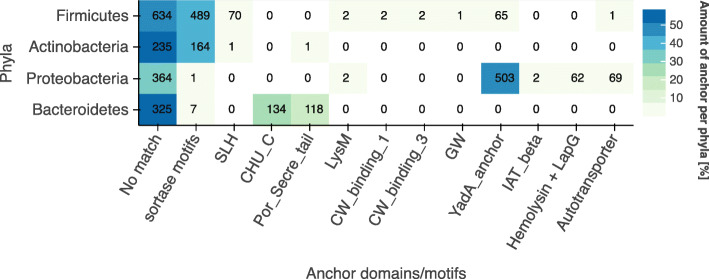
Prevalence of anchoring motifs and domains in FA-like proteins: The heatmap shows the anchor domains and motifs found in the FA-like proteins of the four main phyla. The colors represent the percentage abundance per phyla. The total number of anchors identified is indicated in each cell

To better characterise FA-like proteins we created a list of known anchoring motifs, signals and domains (supplementary table S3). The frequency of each of these different types of anchoring methods is shown in Fig. 4 below. For most of the detected FA-like proteins (1,785 in all phyla), none of the known anchor domains or motifs listed in supplementary table S3 were found (labelled as No match). For those proteins lacking a known anchor we supposed they may be anchored with a transmembrane spanning helix. Searching with TMHMM we found transmembrane regions for only 20% of the FA-like proteins without known anchor domains.

The Gram positive anchor motif is the most prevalent one in the detected FA-like proteins with 668 hits in total, of which 489 FA-like proteins belong to Firmicutes and 164 belong to the Actinobacteria phylum. In addition to the LPxTG motif, the alternative Gram positive anchor motifs LPxTA, NPxTG, LPxGA, LAxTGand NPQTN were included [19].

The S-layer homology domain was found in 72 detected FA-like proteins and again mostly in the generally Gram positive Firmicutes. The anchor domain CHU_C is found in 134 FA-like proteins, all of which belong to the Bacteroidetes phylum. Por_Secre_tail is found in 124 FA-like proteins and mostly in Bacteroidetes. Four proteins with LysM domains (Pfam: PF01476) were detected, two proteins with Choline_bind_1 (Pfam: PF01473), two proteins with Choline_bind_3 (Pfam: PF19127), two with IAT_beta and one with GW domain. In Proteobacteria the YadA anchor (Pfam: PF03895) domain, Autotransporter domain (Pfam: PF03797) and the LapG + HemolysinCabind anchor were the most prevalent. The LapG motif is the protease cleavage site at the N-terminus of RTX adhesins, where the proteins are cleaved by the LapG protease and subsequently inserted into a beta barrel of an outer membrane pore [20]. To be certain to find the LapG cleavage site in RTX adhesins, we additionally searched for the characteristic RTX repeats, HemolysinCabind domain (Pfam: PF00353), at the C-terminus, which is a Type 1 Secretion System signal [21, 22].

We ran the protein subcellular localization predictor PSORTb to identify that 871 of the 3,542 FA-like proteins were predicted to be localised at the cell wall, 547 to be at the outer membrane, 451 to be extracellular and 1,428 of these proteins had no localisation predicted [23]. PSORTb predicts protein localizations based on the amino acid composition, characteristic motifs, signal peptides, transmembrane helices or based on similar proteins with known cellular localizations [24].

### Domain architecture of FA-like proteins

**Fig. 5:**
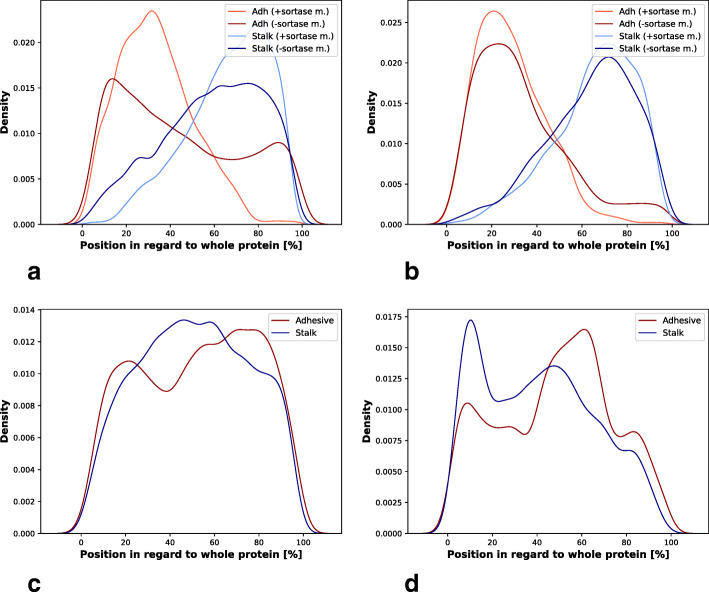
Domain architecture of FA-like proteins split by bacterial phyla: In these density plots the position of adhesive and stalk domains in FA-like proteins of the four main represented taxonomic phyla are plotted. These are **a**) Firmicutes (1248 protein hits), **b**) Actinobacteria (400 protein hits), **c**) Proteobacteria (1001 protein hits) and **d**) Bacteroidetes (581 protein hits). 0% on the x-axes hereby represent the N-termini and 100% the C-termini of the proteins. The data are split between proteins with sortase anchor motif or without for the Gram positive Firmicutes and Actinobacteria phyla

It is generally assumed that, in fibrillar adhesins, adhesive domains are projected away from the bacterial cell surface by the elongated structure composed of stalk domains, promoting its interaction with the host cell. This architecture is similar to multiprotein pili adhesins, where the protein with the binding function is the membrane distal protein of all pili subunits [25]. In fibrillar adhesins the adhesive domain should be at the distal end of all stalk domains compared to the anchor. To investigate this assumption, the domain architectures of the identified FA-like proteins were investigated. In known fibrillar adhesins the adhesive domain is indeed often found at the N-terminus, far away from the cell wall anchoring motif at the C-terminus, e.g. in Cna of *S. aureus* [26, 27]. In order to see where in the protein the adhesive and stalk domains are annotated, their positions were plotted in Fig. 5. The plots are split between the most prevalent phyla of the detected FA-like proteins, which are Firmicutes (35%), Proteobacteria (28%), Bacteroidetes (16%) and Actinobacteria (11%). For Actinobacteria and Firmicutes, we see a strong signal with the adhesive domain found at the N-terminus and the stalk domains found towards the C-terminus of the proteins (Fig. 5a,b). This can be explained by the presence of the sortase anchor motif. The anchor motif is mostly found in Gram positive bacteria, where it gets anchored to the peptidoglycan cell wall. We also see the same pattern in proteins lacking the sortase motif. This is most likely due to some sortase motifs being missed by current search methods. Interestingly, the FA-like proteins without a sortase motif in Firmicutes do show a small peak at the C-terminus, which might point out a different anchor method being used.

In the Gram negative phyla Bacteroidetes and Proteobacteria, the distribution of domains is more variable. For Proteobacteria, the adhesive as well as the stalk domains show a relatively even distribution along the length of the FA-like proteins with a weak preference for adhesive domains at the termini (Fig. 5c). In Bacteroidetes, the stalk and adhesive domain distributions are similar, but, surprisingly, many adhesive domains are found in the middle of the protein (Fig. 5d).

There are known examples of centrally located adhesive domains. One example is adhesin P1 protein in *S. mutans* (also called AgI/II, SpaP, PAc, antigen B), where the adhesive domain Adhesin_P1_N is annotated at the N-terminus and a second adhesive domain GbpC is annotated in the middle of the protein sequence. Adhesin_P1_N folds then to interact with the stalk domains Antigen_C and AgI_II_C2 at the C-terminus, which results in having GbpC at the top of the protein [14]. Adhesin P1 belongs to the antigen I/II family and is known to bind to the saliva on the enamel tooth surface [28].

### Taxonomic overview

**Fig. 6:**
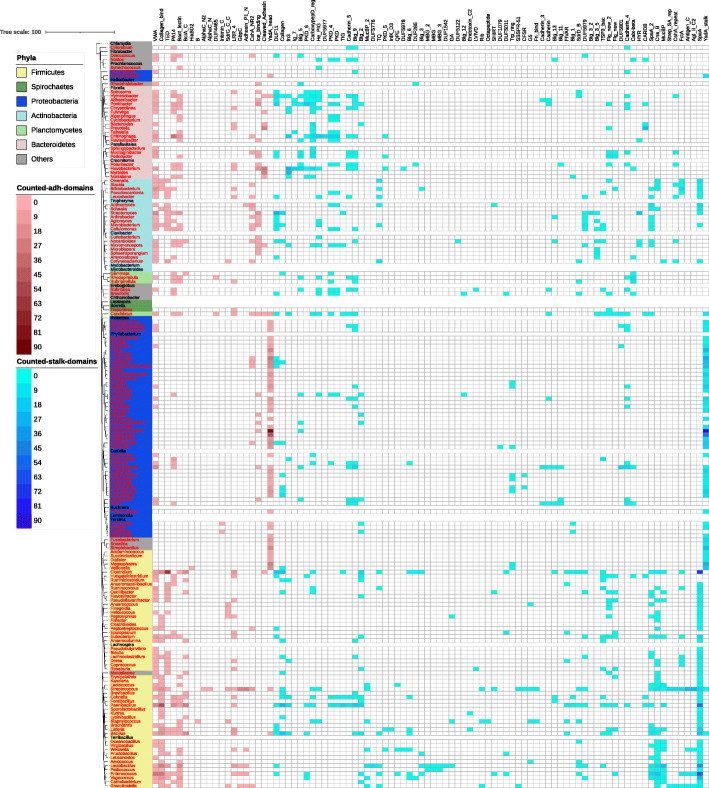
Taxonomic overview of adhesive and stalk domains in FA-like proteins: This taxonomic tree is built with Jalview using the Neighbour Joining algorithm with representative bacterial genera of Firmicutes (yellow), Spirochaetes (dark green), Proteobacteria (dark blue), Actinobacteria (light blue), Planctomycetes (light green), Bacteroidetes (light red) and other phyla (grey). For the genera marked in red text FA-like proteins could be detected. To visualise which adhesive and stalk domains were mainly found in combination in the genera, they are mapped in blue and red respectively

To get a deeper understanding on the origins and evolution of FA-like proteins, we looked at the distribution of adhesive and stalk domains across the bacterial phyla (see Fig. 6). FA-like proteins are widespread across the bacterial tree of life, found in 28 of 56 phyla of the UniProt Reference Proteomes. 14 of the phyla without FA-like proteins have only a single reference proteome, suggesting that the apparent lack of FA-like proteins may be due to lack of sampling of proteomes. Table 1 lists the bacterial reference proteomes with the highest number of FA-like proteins per proteome size; among them we can find the well studied pathogen *Listeria monocytogenes*. The number of FA-like proteins per bacterial reference proteome varies: proteomes with the highest prevalence commonly contain in the order of 10 FA-like proteins, reaching in the case of *Streptobacillus moniliformis* up to 21 FA-like proteins (1.47%).

**Table 1:** Bacterial strains with the highest fraction of FA-like proteins per proteome size

Organism	Proteome ID (UniProt)	Proteome size	FA-like proteins	Fraction [%]
*Streptobacillus moniliformis* (strain ATCC 14647 /	UP000002072	1431	21	1.47
DSM 12112 / NCTC 10651 / 9901)				
*Histophilus somni* (strain 2336)	UP000008543	1971	12	0.61
*Streptococcus sanguinis* (strain SK36)	UP000002148	2269	12	0.53
*Sneathia amnii*	UP000033103	1182	6	0.51
*Listeria monocytogenes* serovar 1/2a (strain ATCC	UP000000817	2844	14	0.49
BAA-679 / EGD-e)				
*Myroides indicus*	UP000295215	2689	13	0.48
*Basilea psittacipulmonis* DSM 24701	UP000028945	1452	7	0.48
*Veillonella criceti*	UP000255367	1926	9	0.47
*Taylorella asinigenitalis* (strain MCE3)	UP000009284	1523	7	0.46
*Haemophilus parasuis* serovar 5 (strain SH0165)	UP000006743	2002	9	0.45
*Mycoplasma* sp. CAG:877	UP000018280	1407	6	0.43
*Luteimonas* sp. H23	UP000308707	3308	14	0.42
*Actinobacillus seminis*	UP000215738	2028	8	0.39
*Veillonella* sp. oral taxon 780 str. F0422	UP000010295	1585	6	0.38
*Weissella ceti*	UP000029079	1338	5	0.37
*Clostridium* sp. CAG:354	UP000018313	1707	6	0.35
*Erboglobus luteus*	UP000244896	2881	10	0.35
*Pseudoscardovia suis*	UP000216454	1732	6	0.35
*Streptococcus gordonii* (strain Challis / ATCC 35105 /	UP000001131	2050	7	0.34
BCRC 15272 / CH1 / DL1 / V288)				
*Opitutaceae bacterium* TSB47	UP000078486	4993	17	0.34

The adhesive and stalk domains in FA-like proteins mapped to the genera in Fig. 6 shows that overall the domain composition of Gram positive Firmicutes and Actinobacteria are similar to each other, but different from the Gram negative Proteobacteria. There are also Gram negative genera in Firmicutes, *Succiniclasticum*, *Acidaminococcus*, *Dialister*, *Veillonella* and *Megasphaera*, whose domain composition are more similar to the Proteobacteria. This underlines the importance of the cell surface composition for the architecture of FA-like proteins. In Proteobacteria the FA-like proteins are mainly built out of YadA_head and YadA_stalk domains. For nearly all of the Firmicutes genera and many Actinobacteria FA-like proteins composed of Collagen_bind, VWA or TED (Pfam: PF08341) in combination with the SpaA stalk domain could be detected. The Gram negative Bacteroidetes use a wider range of domains compared to the Proteobacteria, of which many adhesive domains are the same as in Gram positive bacteria.

There are adhesive and stalk domains found in a wide range of genera, as well as adhesive and stalk domains limited to a few specific genera. VWA, for example, is an adhesive domain widely distributed and found in combination with different stalk domains in Firmicutes, Proteobacteria, Bacteroidetes, Planctomycetes and Actinobacteria. On the other hand, Intimin_C (Pfam: PF07979), seems to be adapted to Proteobacteria as it was only detected in this phylum in combination with the Big_1 (Pfam: PF02369) and Big_2 (Pfam: PF02368) stalk domains.

### Boundary between stalk and adhesive domain definitions

We observed that some of the domains classified as adhesive in our list can also be found in repeats, leading us to question the limits between adhesive and stalk domain functions, and whether they can be interchangeable. Multiple adhesive domains repeated in tandem might form stalks, and stalk domains might evolve adhesion function over time to increase the binding avidity to host cells.

**Fig. 7:**
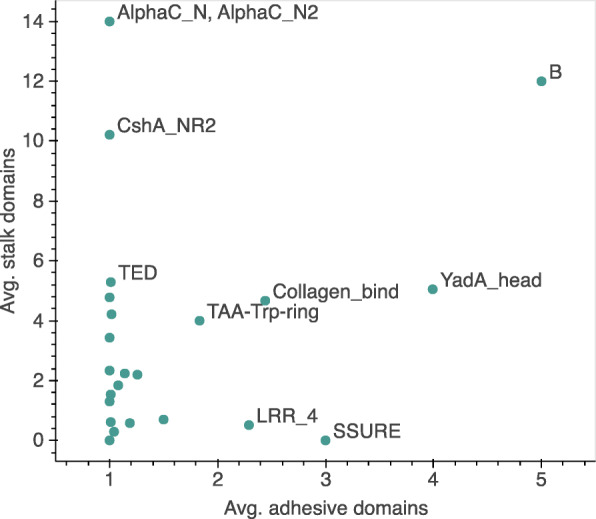
Average of domains found per adhesive domain family: For each adhesive domain family, the average number of adhesive domains is plotted against the average number of stalk domains

In Fig. 7 we can see that most adhesive domains are only found once per protein. Exceptions are SSURE, B (Pfam: PF02216), YadA_head, Collagen_bind and LRR_4 (Pfam: PF12799). Contrary to Collagen_bind and B domains, the SSURE domain is not found in combination with known stalk domains and we hypothesise that it may function as both adhesive and stalk domain. The Pertactin domain is found within the binding region of the *Haemophilus influenzae* Hap adhesin [29], but forms into a beta-helical stalk and therefore might be able to function as a stalk. The Fn_bind (Pfam: PF02986) domain was defined as a stalk domain because it is found in combination with the adhesive domains SdrG_C_C and VWA. However, it is known that Fn_bind also has a binding function and can bind to Fibronectin, e.g. in the Fibronectin-binding proteins of *S. aureus* [30]. Similarly the repeating stalk domain AIDA, which was shown to enhance biofilm formation and autoaggregation in the AIDA-I autotransporter in *E. coli* [31], suggesting that Fn_bind and AIDA are two other examples of dual adhesive and stalk domains.

## Discussion

In this paper we aimed to comprehensively identify FA-like proteins and describe their characteristic domain composition, architecture and taxonomic distribution. Our identification approach is based on Pfam family HMMs of known adhesive and stalk domains, and has resulted in the discovery of many novel FA-like proteins from a wide range of bacterial genera. These proteins include new putative binding proteins in well studied pathogens like *Listeria monocytogenes*, but also in poorly characterised pathogens, like *Sneathia amnii*.

Our results show the various different adhesive and stalk domain combinations that exist in nature and also how frequently they are found. The information can facilitate the identification of new fibrillar adhesins. For instance, when analysing a new FA-like protein, a potential annotated adhesive domain can infer the existence of a specific stalk domain and vice versa. Domain grammar approaches have shown that a knowledge of the domain grammar of proteins can enhance sequence similarity searching [32, 33]. For a representative analysis of the phylogenetic distribution of FA-like proteins we decided to use the UniProt Reference Proteomes sequences only. We expect that more FA-like proteins and more adhesive and stalk domain combinations can be found using the whole UniProtKB database (supplementary figure S3), although this approach may lead to redundancy and unintended biases in our observations. We observed that several adhesive domains can be found in combination with different stalk domains, raising the question whether adhesive domains are able to function with any arbitrary stalk domain. We suggest that this would be an interesting question for experimental studies to address.

One of the more striking results of our domain grammar analysis was the presence of adhesive domains at the N-terminus of Firmicutes FA-like proteins. This preference is largely due to the presence of a C-terminal sortase motif in these proteins. For many of the analysed adhesive domains their relative position in the FA-like proteins stays constant (supplementary figure S4). This observation can be partly explained by the low number of FA-like proteins in which adhesive domains are found, for example in the case of AlphaC_N. There might be further limitations of adhesive domains to be found at either side of the stalk in FA-like proteins, such as effective binding orientation. The Bact_lectin domain is mostly found in the N-terminal third of FA-like proteins (supplementary figure S4) and binds sucrose at the N-terminal end of the adhesive domain in the SraP adhesin [34]. It is questionable whether Bact_lectin domains would be able to function as adhesive domains when found C-terminal to the stalk, but we find an example in a FA-like protein with a Por_Secre_tail anchor. The relative position seems less important for adhesive domains like SdrG_C_C, which binds fibrinogen in a trench formed with the Big_8 domain [35] and which is predominantly found in the middle or C-terminal part of FA-like proteins (supplementary figure S4), and other domains that form tandem repeats such as Collagen_bind, PA14 and VWA. When we investigate Firmicutes proteins without a sortase anchor, we still see this N-terminal preference for adhesive domains. This seems likely due to our weak ability to detect short anchoring motifs. In nearly half of the detected FA-like proteins, no known anchor was found, suggesting novel anchor domains or motifs may yet exist to be discovered.

The investigation of the taxonomic distribution of adhesive and stalk domains show a wide range of different domains in Gram positive bacteria. One reason for this variety might be that the majority of fibrillar adhesins already studied belong to Firmicutes genera, as for example *S. aureus* [36], and subsequently a large number of adhesive domains on our list were first discovered in Firmicutes. Most of the FA-like proteins in the Gram negative Proteobacteria are composed of YadA_head and YadA_stalk domains. A reason for the low diversity of FA-like protein hits in Proteobacteria is a lack of information about fibrillar adhesins in Gram negative bacteria. Many Gram negative autotransporters with adhesion function are known [37], but the repeats and particularly adhesive domains in these proteins are quite poorly defined in Pfam and other domain databases. Another possibility is that Gram negative bacteria might predominantly use pili or Outer Membrane Proteins [38] for the adhesion to host cells and therefore possess less fibrillar adhesins. The taxonomic investigation furthermore demonstrates that there are domains which are found to be common between widely diverged phyla such as Firmicutes and Actinobacteria. But it also shows that there are domains which are only found in a specific phylum such as Intimin_C. In the case of Intimin_C, we know from its structure that it is distantly related to other C-type lectin domains. In these cases of phylogenetically restricted domains, it will be interesting to understand to what extent they have evolved novel binding specificities.

In our results, we describe that the adhesive domain SSURE is not found in combination with other known stalk domains, and that it is found on average in 3 copies per protein. We additionally know that SSURE is tandemly repeated. This leads to the hypothesis that some adhesive domains might be able to function as the stalk itself. Several adhesive domains are indeed found repeated. A reason for repeating adhesive domains could be to increase the avidity of binding to the host cells. Thus, duplications of these domains may lead to both extending the stalk length and increasing binding efficiency. In particular, when the host ligand is a collagen, the adhesive domain might bind to different positions simultaneously on the host ligand. The adhesive domain Collagen_bind binds collagen with the very specific collagen hug mechanism which requires the supra-domain of Big_8 domain N-terminal to the Collagen_bind domain. Therefore, it would be interesting to find out why Collagen_bind occurs in repeats and whether all repeats contribute to the adhesion process.

The greatest limitation of our FA-like protein discovery process is that it requires a protein to contain a combination of known stalk and adhesive domains. There are undoubtedly numerous fibrillar adhesive proteins missed by our approach. There are several reasons for these missed proteins. Firstly, there may be FA-like proteins that have missing domain annotations for either the stalk or adhesive domain or both. This could be because there is no domain family represented within Pfam currently. A second possibility is that the current Pfam model misses true examples of the stalk or adhesive domain. And thirdly, simply that there are adhesive and stalk domains we did not yet identify. An estimation of the completeness of the stalk domain list for known adhesive domains is given in supplementary table S4. Nevertheless, the information about fibrillar adhesins gained in this study will be important for further improvements in future identification of these proteins.

## Conclusions

This study presents to our knowledge the most comprehensive characterisation of fibrillar adhesins to date. We have used a protein domain based screen to identify over 3,000 fibrillar adhesin-like proteins and characterised their domain content, architecture and taxonomic distribution. The results underline the complexity of fibrillar adhesins, with highly variable domain organisations as well as with various different domain combinations found in nature. The detected FA-like proteins are widespread across the bacterial taxonomic tree. The set of FA-like proteins and associated stalk and adhesive domains identified in this study set a foundation stone to discover further fibrillar adhesins.

## Methods

### Collection of stalk, adhesive and anchor domains

We searched in the literature for experimentally confirmed bacterial adhesins, for example by a keyword search for adhesins in PubMed. We analysed the domain architecture of the relevant proteins and identified the adhesive domains by searching for the binding regions. We also identified numerous adhesive domains through investigation of structures in the Protein Data Bank. In each case we identified the relevant entries in the Pfam database and recorded the relevant identifiers.

Stalk domains were detected by searching for tandem repeat domains in the Pfam database, whereby repeats are counted as tandem repeats when the sequence separation between two domains from the same Pfam family is shorter than 35 residues. The further putative stalk domain families were checked manually in Pfam to ensure their description, species distribution and domain architecture matched the stalk domain definition. Domain families found only in eukaryotes, and non-surface and enzymatic proteins, were excluded.

Tandem sequence repeats were identified in proteins without known Pfam annotations with the T-REKS tool using 70% sequence identity and length between 50-200 residues as parameters [39]. Six new Pfam families were built (Pfam: PF19403 - PF19408) by first clustering sequence repeats with BLAST and then iterating alignments of the largest clusters [40].

### Identification of fibrillar adhesin-like proteins

In this study all bacterial proteins from the UniProt Reference Proteomes (release 2020_03) were used [12]. The 7,581 bacterial reference proteomes encompass 28,086,379 proteins. We searched with the identified adhesive and stalk Pfam domain HMMs against the bacterial protein sequences using the HMMER tool (version 3.1b2) with the gathering (GA) threshold option [41]. Overlapping annotations were excluded based on the hmmsearch annotation score. We identified proteins with at least one known adhesive domain (supplementary table S2) and one known stalk domain (supplementary table S1) and constructed a fasta sequence database for the detected proteins. The matches from this query were considered as FA-like proteins and were used for the majority of analyses. For comparison, FA-like proteins were also searched for in the whole UniProtKB using the same approach.

### Characterization of fibrillar adhesin-like proteins

The number of stalk domains per protein in combination with one adhesive domain were counted, regardless of whether the stalk domains belonged to the same Pfam family or not. The frequency of adhesive and stalk domains as well as the combinations of adhesive and stalk domains and different stalk domains found on the same FA-like proteins were counted, whereby each domain was only counted once even if multiple domains of the same family were annotated on the protein.

For the generation of the density plots representing the domain positions within the proteins, the protein length and the envelope domain location information were used from the HMMER search output file. The fractional start and end positions for the domains were calculated. Furthermore, sortase motifs were identified by regular expression in the C-terminal 50 amino acids of the FA-like protein sequences. For the analysis of the domain architecture between the different phyla, the taxonomic phyla were mapped to the UniProt protein identifier by using Retrieve/ID mapping from the UniProt website (https://www.uniprot.org/uploadlists/).

### Identification of cell anchoring mechanisms of FA-like proteins

To find out how FA-like proteins are attached to the bacterial cell, HMMs of known anchor domains were downloaded from Pfam. We used the following models: Gram_pos_anchor (Pfam: PF00746), SLH (Pfam: PF00395), CHU_C (Pfam: PF13585), Por_Secre_Tail (Pfam: PF18962), LysM (Pfam: PF01476), Choline_bind_1 (Pfam: PF01473), Choline_bind_2 (Pfam: PF19085), Choline_bind_3 (Pfam: PF19127), YadA_anchor (Pfam: PF03895) IAT_beta (Pfam: PF11924), GW (Pfam: PF13457), PG_binding_1 (Pfam: PF01471), PG_binding_2 (Pfam: PF08823) and PG_binding_3 (Pfam: PF09374) [42]. By HMMER (version 3.2.1) hmmsearch using each profile’s Gathering (GA) threshold the FA-like protein sequences were searched for the above listed anchor domains [41]. To improve the search sensitivity for Gram positive anchor motifs, we carried out additional regular expression searches for the canonical sequence motifs LPxTA and LPxTG as well as for the alternative anchor motifs NPxTG, LAxTG, NPQTN and LPxGA. To be considered a match we also required these regular expression matches to occur within the C-terminal 50 amino acids of the sequence. If these sortase motif protein hits were missed by the hmmsearch for the Gram_pos_anchor, the identifiers were added and are displayed together as sortase motifs in the bar plots. It was shown that RTX adhesins can be inserted into a beta barrel of an outer membrane pore with its N-terminus [20]. Shuaiqi Guo et al. (2017) aligned the N-terminus of ten RTX adhesins of different Gram negative bacteria and showed that an alpha-helical element (xxIQxAIAA), followed by a short coil motif (GxDPT) and by the LapG protease cleavage motif (uAAG, where the u is a threonine or proline residue) are well aligned in these proteins [20]. By using their ten representative RTX adhesin sequences the alignment was reproduced and an HMM was built, which yielded 37 hits in all 3,542 FA-like protein sequences. But even in known RTX adhesins, like MhLap of *Marinobacter hydrocarbonoclasticus* (UniProt: H8W6K8), the described secondary structure motifs were not found so we only searched for the protease cleavage motif AAG within the first 150 amino acids in the FA-like protein sequences. To increase the probability that the found hits are indeed RTX adhesins, we additionally searched for the HemolysinCabind domain, which is characteristic for the RTX adhesins. In total 62 LapG cleavage motifs and HemolysinCabind domains could be found together in a single protein. In 31 of these 62 FA-like protein hits the short coil motif GxDPT could be found, whereas the alpha-helical element motif xxIQxAIAA was only found in two of these proteins. For comparison, the PSORTb webserver (version 3.0.2) was used to predict the localization of the 3,542 FA-like proteins [23].

### Taxonomic analysis

For taxonomic analysis, additional to the phyla, the genera were also mapped by using Retrieve/ID mapping from the UniProt website. The 16s rRNA sequences of representative bacterial genera and of genera with a minimum of 5 detected FA-like proteins were aligned in JalView using Muscle with the default settings [43, 44]. Afterwards a Neighbour Joining based tree was calculated with JalView by using the BLOSUM62 matrix for measuring the distance of the alignments [43]. For the visualisation of the tree iTOL was used [45]. For each genera the adhesive and stalk domains of FA-like proteins were counted and mapped to the tree. Again, stalk domain repeats on the same FA-like protein were counted as one occurrence.

### Searching for adhesive and stalk repeats

Protein sequences containing known adhesive domains were downloaded from Pfam version 33.1 [42]. Proteins were further filtered to be from bacterial species and a minimum of 500 amino acids long, and searched against Pfam using the HMMER tool (version 3.2.1) with the gathering (GA) threshold option [41]. Overlapping annotations were again excluded based on the hmmsearch annotation score. The number of stalk and adhesive domain annotations per protein were counted and the average was calculated per adhesive domain family.

### Investigation of completeness of stalk domain list

The HMMER search results of the long bacterial proteins with known adhesive domains against the Pfam database from the “Searching for adhesive and stalk repeats” section were used again. Proteins with known stalk domains were counted as well as proteins without any Pfam domain annotation, except for the anchor domains listed in supplementary table S3, the signal domains YSIRK_signal (Pfam: PF04650), ESPR (Pfam: PF13018) and the TAT_signal (Pfam: PF10518) and the domains in known supra domains.

### Analysing the relative position of the adhesive domains within FA-like proteins

For each adhesive domain found in the FA-like proteins we calculated if its mid point is within the N-terminal, middle or C-terminal third of the FA-like protein. We then calculated the percentage of occurrence in each of the three relative positions for each adhesive domain. The results are presented in the heatmap in supplementary figure S4.

## Supplementary Information

**Additional file 1** Supplementary figures and tables.

## Data Availability

The Pfam database (version 33.1) used for this study can be accessed from http://ftp.ebi.ac.uk/pub/databases/Pfam/releases/Pfam33.1/. The UniProt Reference Proteomes (release 2020_03) are available under https://ftp.uniprot.org/pub/databases/uniprot/previous_releases/release-2020_03/knowledgebase/knowledgebase2020_03.tar.gz. Accession numbers of the stalk, adhesive and anchor domain families used in this study are indicated in supplementary tables S1, S2 and S3, respectively. The FA-like protein dataset generated during the current study is available in the GitHub repository https://github.com/VivianMonzon/FA-like_proteins.

## Abbreviations

FA-like: Fibrillar adhesin-like
PG: Peptidoglycan
OM: Outer membrane
GO: Gene ontology
DLL: Dock lock latch
HMM: Hidden Markov model
GA threshold: Gathering threshold
