# Supplementary material for: Discovery of fibrillar adhesins across bacterial species
Source: BMC Genomics. 2021 Jul 18;22:550. doi: 10.1186/s12864-021-07586-2 (PMC8286594; doi:10.1186/s12864-021-07586-2)
Supplement: Supplementary file 1 — Additional file 1 Supplementary figures and tables. [file 12864_2021_7586_MOESM1_ESM.pdf]

Supplementary

Table S1: Stalk domains identified and used in this study

Name	Pfam ID	Pfam Clan	Clan ID
Rib	PF08428	E-set	CL0159
Big_1	PF02369	E-set	CL0159
Big_2	PF02368	E-set	CL0159
Big_3	PF07523	E-set	CL0159
Big_3_5	PF16640	E-set	CL0159
Big_6	PF17936	E-set	CL0159
Big_9	PF17963	E-set	CL0159
DUF5011	PF16403	E-set	CL0159
DUF11	PF01345	E-set	CL0159
PKD	PF00801	E-set	CL0159
He_PIG	PF05345	E-set	CL0159
Cadherin_4	PF17803	E-set	CL0159
Cadherin_5	PF17892	E-set	CL0159
CARDB	PF07705	E-set	CL0159
Calx-beta	PF03160	E-set	CL0159
TIG	PF01833	E-set	CL0159
Cadherin	PF00028	E-set	CL0159
fn3	PF00041	E-set	CL0159
Big_13	PF19077	E-set	CL0159
Big_12	PF19078	E-set	CL0159
Big_11	PF18200	E-set	CL0159
Big_5	PF13205	E-set	CL0159
Big_3_2	PF12245	E-set	CL0159
HYR	PF02494	E-set	CL0159
InlK_D3	PF18981	E-set	CL0159
PKD_4<br>Cadherin_3	PF18911<br>PF16184	E-set<br>E-set	CL0159<br>CL0159
PKD_5	PF19406	E-set	CL0159
PKD_6	PF19408	E-set	CL0159

SHIRT	PF18655	Ubiquitin	CL0072
Flg_new	PF09479	Ubiquitin	CL0072
MucBP	PF06458	Ubiquitin	CL0072
SSSPR-51	PF18877	Ubiquitin	CL0072
MucBP_2	PF17965	Ubiquitin	CL0072
Flg_new_2	PF18998	Ubiquitin	CL0072
DUF1542	PF07564	B_GA	CL0598
GA	PF01468	B_GA	CL0598
FIVAR	PF07554	B_GA	CL0598
MBG	PF17883	MBG	CL0682
MBG_2	PF18676	MBG	CL0682
MBG_3	PF18887	MBG	CL0682
FctA	PF12892	Transthyretin	CL0287
SpaA	PF17802	Transthyretin	CL0287
Cna_B	PF05738	Transthyretin	CL0287
SdrD_B	PF17210	Transthyretin	CL0287
CarboxypepD_reg	PF13620	Transthyretin	CL0287
SpaA_2	PF19403	Transthyretin	CL0287
DUF5979	PF19407	Transthyretin	CL0287
G5	PF07501	G5	CL0593
LVIVD	PF08309	Beta_propeller	CL0186
DUF5122	PF17164	Beta_propeller	CL0186
SBBP	PF06739	Beta_propeller	CL0186
Ig_7	PF19081	Ig	CL0011
I-set	PF07679	Ig	CL0011
TSP3_bac	PF18884	TSP3	CL0689
DUF285	PF03382	LRR	CL0022
SlpA	PF03217	No clan	No clan
AgI_II_C2	PF17998	No clan	No clan
Collagen	PF01391	No clan	No clan
Fn_bind	PF02986	No clan	No clan
CshA_repeat	PF19076	No clan	No clan
Antigen_C	PF16364	No clan	No clan

TQ	PF18202	No clan	No clan
YadA_stalk	PF05662	No clan	No clan
Endotoxin_C2	PF18449	No clan	No clan
Trp_ring	PF18669	No clan	No clan
Strep_SA_rep	PF06696	No clan	No clan
DUF5801	PF19116	No clan	No clan
DUF1079	PF06435	No clan	No clan
Octapeptide	PF03373	No clan	No clan
DUF5776	PF19087	No clan	No clan
QPE	PF18874	No clan	No clan
CFSR	PF19079	No clan	No clan
DUF5977	PF19404	No clan	No clan
DUF5978	PF19405	No clan	No clan
AIDA	PF16168	Pec_lyase-like	CL0268
PATR	PF12951	Pec_lyase-like	CL0268
Fil_haemagg	PF05594	Pec_lyase-like	CL0268
Fil_haemagg_2	PF13332	Pec_lyase-like	CL0268
Beta_helix_3	PF18889	Pec_lyase-like	CL0268
Beta_helix	PF13229	Pec_lyase-like	CL0268
DUF5649	PF18886	Pec_lyase-like	CL0268

Table S2: Adhesive domains used in this study

**Name**	**Pfam ID**	**Pfam Clan**	**Clan ID**	**Host ligand type**	**Example (UniProt ID)**	**Ref.**
Bact_lectin	PF18483	Concanavalin	CL0004	Carbohydrate	G8PFB6	[1]
Cleaved_Adhesin	PF07675	Concanavalin	CL0004	Carbohydrate	Q51817	[2, 3]

SdrG_C_C	PF10425	Adhesin	CL0204	Protein	Q2G0L4	[4]
Collagen_bind	PF05737	Adhesin	CL0204	Protein	Q53654	[5]
FimH_man-bind	PF09160	Adhesin	CL0204	Carbohydrate	P08191	[6]
PA14	PF07691	PA14	CL0301	Carbohydrate	P13423	[7, 8]
LRR_4	PF12799	LRR	CL0022	Protein	P0DJM0	[9]
B (SpAB)	PF02216	B_GA	CL0598	Protein	P02976	[10]
Intimin_C	PF07979	C_Lectin	CL0056	Protein	P43261	[11]
VWA	PF00092	vWA-like	CL0128	Protein	A0A242JZH4	[12]
BclA_C	PF18573	C1q_TNF	CL0100	Protein	Q6I1X8	[13]
FadA	PF09403	OML zippers	CL0590	Protein	Q5I6B0	[14]
CshA_NR2	PF18651	No clan	No clan	Protein	Q54194	[15]
YadA_head	PF05658	No clan	No clan	Protein	P31489	[16]
AlphaC_N	PF08829	No clan	No clan	Protein	Q8E1C4	[17]
AlphaC_N2	PF17480	No clan	No clan	Protein	Q8E1C4	[17]
SabA_adhesion	PF18304	No clan	No clan	Carbohydrate	O25840	[18]
SSURE	PF11966	No clan	No clan	Protein	Q8DRK2	[19]
TED	PF08341	No clan	No clan	Protein	Q81XH9	[20]
Adhesin_P1_N	PF18652	No clan	No clan	Carbohydrate	P11657	[21]
GbpC	PF08363	No clan	No clan	Carbohydrate	P17953	[22]
Ice_binding	PF11999	No clan	No clan	Ice crystals	H7FWB6	[23]
HiaBD2	PF15403	No clan	No clan	Protein	Q48152	[24]
Pertactin	PF03212	Pec_lyase-like	CL0268	Protein	P45387	[25]
TAA-Trp-ring	PF15401	No clan	No clan	Protein	P71401	[26]
Sgo0707_N1	PF18873	No clan	No clan	Protein	A8AW49	[27]
BspA_v	PF18220	No clan	No clan	Protein	A0A380IYG9	[28]

Table S3: Anchor domains used in this study

**Name**	**Pfam ID**	**Notes**	**Ref.**
Gram_pos_anchor	PF00746	Covalent binding to peptidoglycan	[29]
Choline_bind_1	PF01473	Choline binding repeats (CW)	[30]
Choline_bind_2	PF19085	Choline binding repeats (CW)	[30]

CW_binding_3	PF19127	Choline binding repeats (CW)	[30]
LysM	PF01476	Lysin motif; Anchoring to Peptidoglycan	[31]
GW	PF13457	Gly-Trp dipeptide; Non-covalent binding to lipoteichoic acids	[32]
SLH	PF00395	S-layer homology; Non-covalent to cell wall polysaccharides	[33]
CHU_C	PF13585	Covalent linkage to A-LPS; T9SS	[34]
Por_Secre_Tail	PF18962	Equivalent to CHU_C	[34]
YadA_anchor	PF03895	Four beta strands; beta barrel in outer membrane	[35]
IAT_beta	PF11924	Beta barrel in outer membrane	[36]
PG_binding_1	PF01471	Potential peptidoglycan binding	[37]
PG_binding_2	PF08823	Potential peptidoglycan binding	[37]
PG_binding_3	PF09374	Potential peptidoglycan binding	[37]
Autotransporter	PF03797	Beta barrel in outer membrane	[38]

Table S4: Investigation of completeness of the stalk domain list. Bacterial proteins in Pfam with a length of minimum 500 residues were counted per adhesive domain and listed in the second column 'Long proteins'. The coverage of long proteins with known stalk domains are counted in the third column 'With known stalk'. The fourth column represents the number of long proteins, without any Pfam annotation and therefore proteins with potential uncharacterized stalk domains.

**Name**	**Long proteins**	**With known stalk**	**W/o annotations**
Bact_lectin	396	165	129
Cleaved_Adhesin	206	103	75
SdrG_C_C	51	38	13
Collagen_bind	399	365	33
PA14	2915	303	318
LRR_4	527	88	133
B (SpAB)	1	1	0
Intimin_C	2	2	0
VWA	4563	490	775
DUF4465	39	3	15
BclA_C	28	25	3

CshA_NR2	28	25	3
YadA_head	1074	877	150
AlphaC_N	1	1	0
AlphaC_N2	1	1	0
SabA_adhesion	6	0	0
SSURE	11	0	11
TED	266	243	19
Adhesin_P1_N	36	26	10
GbpC	65	31	33
Ice_binding	198	133	41

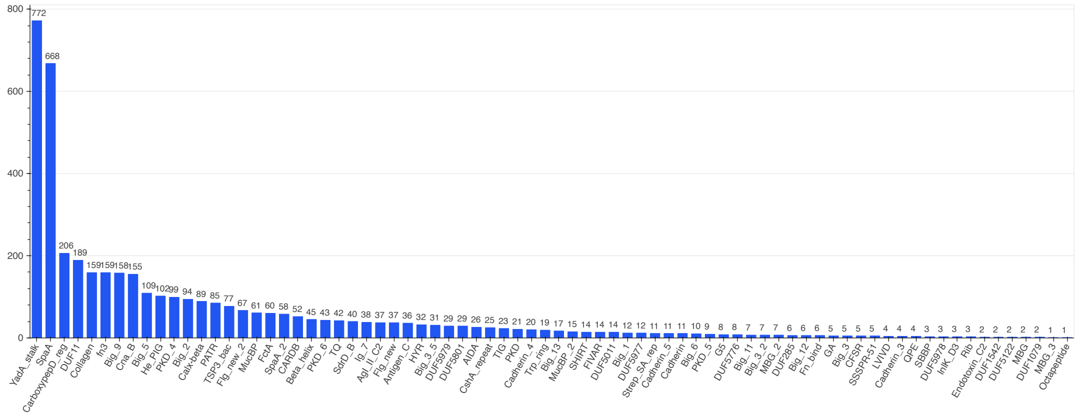

Figure S1: Frequency of stalk domains in combination with adhesive domains: Number of FA-like proteins in which the stalk domains were detected.

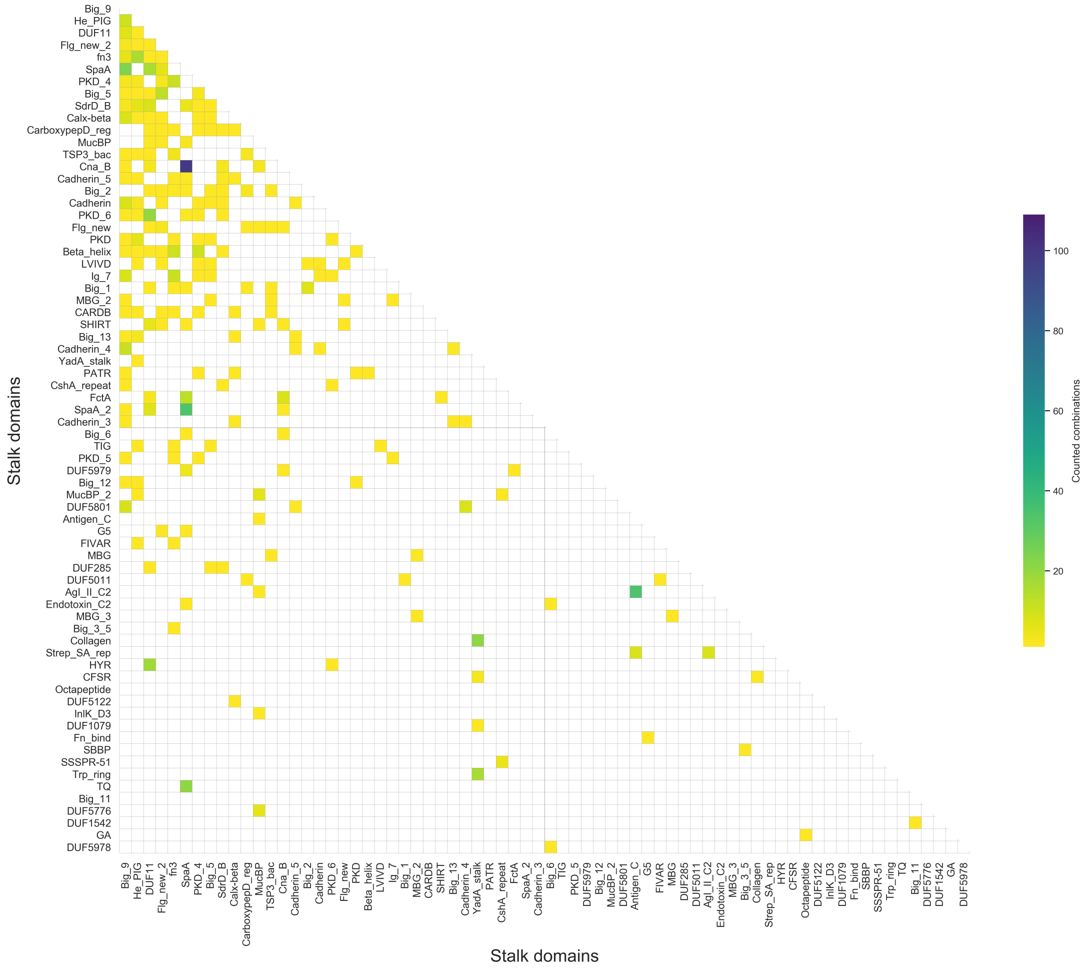

Figure S2: Domain combinations of stalk domains: Heatmap showing the co-occurrence of stalk domains on the same FA-like protein.

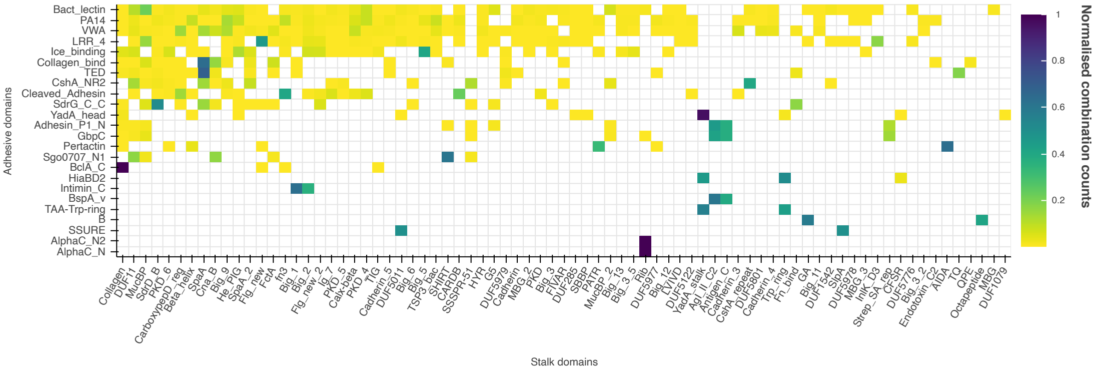

Figure S3: Domain combinations of adhesive and stalk domains found in UniProtKB: Compared to figure 3, this heatmap is based on detected FA-like proteins not only found in the reference proteomes, but the whole UniProtKB.

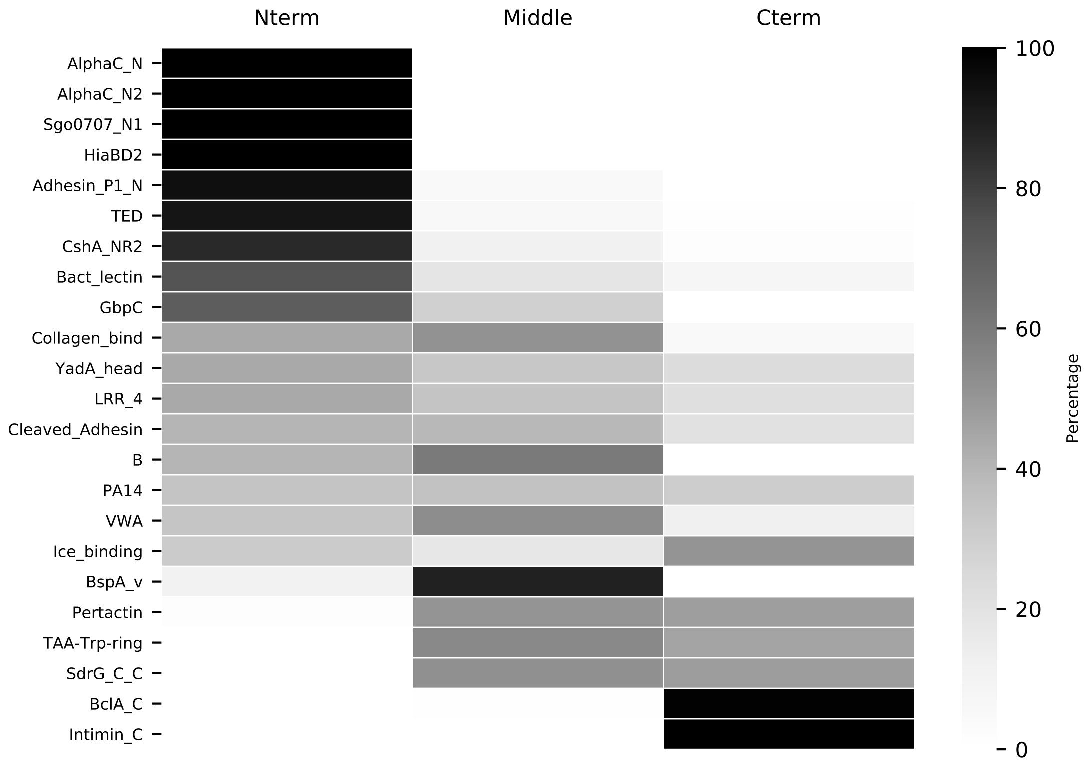

Figure S4: Preferential adhesive domain position within the FA-like proteins: This heatmap shows the varying position within the FA-like proteins for the adhesive domains found in combination with stalk domains. The FA-like proteins are split between the N-terminal (Nterm), middle (Middle) or C-terminal (Cterm) third of each protein for representing the preferential adhesive domain position.

References

[1] Yang YH, Jiang YL, Zhang J, Wang L, Bai XH, Zhang SJ, et al. Structural Insights into SraP-Mediated Staphylococcus aureus Adhesion to Host Cells. PLoS Pathogens. 2014 Jun;10(6):e1004169. Available from: https://dx.plos.org/10.1371/journal.ppat.1004169.

[2] Li N, Yun P, Nadkarni MA, Ghadikolaee NB, Nguyen KA, Lee M, et al. Structure determination and analysis of a haemolytic gingipain adhesin domain from Porphyromonas gingivalis: Structure of a P. gingivalis gingipain adhesin domain. Molecular Microbiology. 2010 Mar;76(4):861–873. Available from: http://doi.wiley.com/10.1111/j.1365-2958.2010.07123.x.

[3] Ganuelas LA, Li N, Yun P, Hunter N, Collyer CA. The lysine gingipain adhesin domains from Porphyromonas gingivalis interact with erythrocytes and albumin: Structures correlate to function. European Journal of Microbiology & Immunology. 2013 Sep;3(3):152–162.

[4] Ponnuraj K, Bowden MG, Davis S, Gurusiddappa S, Moore D, Choe D, et al. A “dock, lock, and latch” Structural Model for a Staphylococcal Adhesin Binding to Fibrinogen. Cell. 2003 Oct;115(2):217–228. Available from: https://linkinghub.elsevier.com/retrieve/pii/S0092867403008092.

[5] Zong Y, Xu Y, Liang X, Keene DR, Höök A, Gurusiddappa S, et al. A ‘Collagen Hug’ Model for Staphylococcus aureus CNA binding to collagen. The EMBO Journal. 2005 Dec;24(24):4224–4236. Available from: http://emboj.embopress.org/cgi/doi/10.1038/sj.emboj.7600888.

[6] Hung CS, Bouckaert J, Hung D, Pinkner J, Widberg C, DeFusco A, et al. Structural basis of tropism of Escherichia coli to the bladder during urinary tract infection: FimH mannose-binding pocket. Molecular Microbiology. 2002 May;44(4):903–915. Available from: http://doi.wiley.com/10.1046/j.1365-2958.2002.02915.x.

[7] Vance TDR, Guo S, Assaie-Ardakany S, Conroy B, Davies PL. Structure and functional analysis of a bacterial adhesin sugar-binding domain. PLOS ONE. 2019 Jul;14(7):e0220045. Available from: https://dx.plos.org/10.1371/journal.pone.0220045.

[8] Rigden DJ, Mello LV, Galperin MY. The PA14 domain, a conserved all-β domain in bacterial toxins, enzymes, adhesins and signaling molecules. Trends in Biochemical Sciences. 2004 Jul;29(7):335–339. Available from: https://linkinghub.elsevier.com/retrieve/pii/S0968000404001227.

[9] Schubert WD, Urbanke C, Ziehm T, Beier V, Machner MP, Domann E, et al. Structure of Internalin, a Major Invasion Protein of Listeria monocytogenes, in Complex with Its Human Receptor E-Cadherin. Cell. 2002 Dec;111(6):825–836. Available from: https://linkinghub.elsevier.com/retrieve/pii/S0092867402011364.

[10] Graille M, Stura EA, Corper AL, Sutton BJ, Taussig MJ, Charbonnier JB, et al. Crystal structure of a Staphylococcus aureus protein A domain complexed with the Fab fragment of a human IgM antibody: Structural basis for recognition of B-cell receptors and superantigen activity. Proceedings of the National Academy of Sciences. 2000 May;97(10):5399–5404. Available from: http://www.pnas.org/cgi/doi/10.1073/pnas.97.10.5399.

[11] Luo Y, Frey EA, Pfuetzner RA, Creagh AL, Knoechel DG, Haynes CA, et al. Crystal structure of enteropathogenic Escherichia coli intimin–receptor complex. Nature. 2000 Jun;405(6790):1073–1077. Available from: http://www.nature.com/articles/35016618.

[12] Whittaker CA, Hynes RO. Distribution and Evolution of von Willebrand/Integrin A Domains: Widely Dispersed Domains with Roles in Cell Adhesion and Elsewhere. Molecular Biology of the Cell. 2002 Oct;13(10):3369–3387. Available from: https://www.molbiolcell.org/doi/10.1091/mbc.e02-05-0259.

[13] Kirchdoerfer R, Herrin B, Han B, Turnbough C, Cooper M, Wilson I. Variable Lymphocyte Receptor Recognition of the Immunodominant Glycoprotein of Bacillus anthracis Spores. Structure. 2012 Mar;20(3):479–486. Available from: https://linkinghub.elsevier.com/retrieve/pii/S0969212612000172.

[14] Nithianantham S, Xu M, Yamada M, Ikegami A, Shoham M, Han YW. Crystal Structure of FadA Adhesin from Fusobacterium nucleatum Reveals a Novel Oligomerization Motif, the Leucine Chain. Journal of Biological Chemistry. 2009 Feb;284(6):3865–3872. Available from: https://linkinghub.elsevier.com/retrieve/pii/S002192581981801X.

[15] Back CR, Sztukowska MN, Till M, Lamont RJ, Jenkinson HF, Nobbs AH, et al. The Streptococcus gordonii Adhesin CshA Protein Binds Host Fibronectin via a Catch-Clamp Mechanism. The Journal of Biological Chemistry. 2017;292(5):1538–1549.

[16] Nummelin H, Merckel MC, Leo JC, Lankinen H, Skurnik M, Goldman A. The Yersinia adhesin YadA collagen-binding domain structure is a novel left-handed parallel β-roll. The EMBO Journal. 2004 Feb;23(4):701–711. Available from: http://emboj.embopress.org/cgi/doi/10.1038/sj.emboj.7600100.

[17] Bolduc GR, Madoff LC. The group B streptococcal alpha C protein binds α 1 β 1-integrin through a novel KTD motif that promotes internalization of GBS within human epithelial cells. Microbiology. 2007 Dec;153(12):4039–4049. Available from: https://www.microbiologyresearch.org/content/journal/micro/10.1099/mic.0.2007/009134-0.

[18] Pang SS, Nguyen STS, Perry AJ, Day CJ, Panjikar S, Tiralongo J, et al. The Three-dimensional Structure of the Extracellular Adhesion Domain of the Sialic Acid-binding Adhesin SabA from Helicobacter pylori. Journal of Biological Chemistry. 2014 Mar;289(10):6332–6340. Available from: https://linkinghub.elsevier.com/retrieve/pii/S0021925820444564.

[19] Bumbaca D, LittleJohn JE, Nayakanti H, Rigden DJ, Galperin MY, Jedrzejas MJ. Sequence Analysis and Characterization of a Novel Fibronectin-Binding Repeat Domain from the Surface of *Streptococcus pneumoniae*. OMICS: A Journal of Integrative Biology. 2004 Dec;8(4):341–356. Available from: http://www.liebertpub.com/doi/10.1089/omi.2004.8.341.

[20] Miller OK, Banfield MJ, Schwarz-Linek U. A new structural class of bacterial thioester domains reveals a slipknot topology: Thioester Domains. Protein Science. 2018 Sep;27(9):1651–1660. Available from: http://doi.wiley.com/10.1002/pro.3478.

[21] Heim KP, Crowley PJ, Long JR, Kailasan S, McKenna R, Brady LJ. An intramolecular lock facilitates folding and stabilizes the tertiary structure of Streptococcus mutans adhesin P1. Proceedings of the National Academy of Sciences. 2014 Nov;111(44):15746–15751. Available from: http://www.pnas.org/cgi/doi/10.1073/pnas.1413018111.

[22] Sato Y, Yamamoto Y, Kizaki H. Cloning and sequence analysis of the gbpC gene encoding a novel glucan-binding protein of Streptococcus mutans. Infection and Immunity. 1997 Feb;65(2):668–675.

[23] Vance TDR, Bayer□Giraldi M, Davies PL, Mangiagalli M. Ice□binding proteins and the 'domain of unknown function' 3494 family. The FEBS Journal. 2019 Mar;286(5):855–873. Available from: https://onlinelibrary.wiley.com/doi/abs/10.1111/febs.14764.

[24] Meng G, St Geme JW, Waksman G. Repetitive Architecture of the Haemophilus influenzae Hia Trimeric Autotransporter. Journal of Molecular Biology. 2008 Dec;384(4):824–836. Available from: https://linkinghub.elsevier.com/retrieve/pii/S0022283608012606.

[25] Meng G, Spahich N, Kenjale R, Waksman G, St Geme JW. Crystal structure of the *Haemophilus influenzae* Hap adhesin reveals an intercellular oligomerization mechanism for bacterial aggregation: Structural framework for bacterial aggregation. The EMBO Journal. 2011 Sep;30(18):3864–3874. Available from: http://emboj.embopress.org/cgi/doi/10.1038/emboj.2011.279.

[26] Edwards TE, Phan I, Abendroth J, Dieterich SH, Masoudi A, Guo W, et al. Structure of a Burkholderia pseudomallei Trimeric Autotransporter Adhesin Head. PLoS ONE. 2010 Sep;5(9):e12803. Available from: https://dx.plos.org/10.1371/journal.pone.0012803.

[27] Nylander □, Svensäter G, Senadheera DB, Cvitkovitch DG, Davies JR, Persson K. Structural and Functional Analysis of the N-terminal Domain of the Streptococcus gordonii Adhesin Sgo0707. PLoS ONE. 2013 May;8(5):e63768. Available from: https://dx.plos.org/10.1371/journal.pone.0063768.

[28] Rego S, Heal TJ, Pidwill GR, Till M, Robson A, Lamont RJ, et al. Structural and Functional Analysis of Cell Wall-anchored Polypeptide Adhesin BspA in Streptococcus agalactiae. Journal of Biological Chemistry. 2016 Jul;291(31):15985–16000. Available from: https://linkinghub.elsevier.com/retrieve/pii/S0021925820424263.

[29] Ton-That H, Liu G, Mazmanian SK, Faull KF, Schneewind O. Purification and characterization of sortase, the transpeptidase that cleaves surface proteins of Staphylococcus aureus at the LPXTG motif. Proceedings of the National Academy of Sciences of the United States of America. 1999 Oct;96(22):12424–12429.

[30] García JL, Sánchez-Beato AR, Medrano FJ, López R. Versatility of Choline-Binding Domain. Microbial Drug Resistance. 1998 Jan;4(1):25–36. Available from: http://www.liebertpub.com/doi/10.1089/mdr.1998.4.25.

[31] Bateman A, Bycroft M. The structure of a LysM domain from E. coli membrane-bound lytic murein transglycosylase D (MltD) 1 1Edited by P. E. Wight. Journal of Molecular Biology. 2000 Jun;299(4):1113–1119. Available from: https://linkinghub.elsevier.com/retrieve/pii/S0022283600937781.

[32] Marino M, Banerjee M, Jonquières R, Cossart P, Ghosh P. GW domains of the Listeria monocytogenes invasion protein InlB are SH3-like and mediate binding to host ligands. The EMBO journal. 2002 Nov;21(21):5623–5634.

[33] Mesnage S, Fontaine T, Mignot T, Delepierre M, Mock M, Fouet A. Bacterial SLH domain proteins are non-covalently anchored to the cell surface via a conserved mechanism involving wall polysaccharide pyruvylation. The EMBO journal. 2000 Sep;19(17):4473–4484.

[34] Veith PD, Glew MD, Gorasia DG, Reynolds EC. Type IX secretion: the generation of bacterial cell surface coatings involved in virulence, gliding motility and the degradation of complex biopolymers: The Type IX secretion system. Molecular Microbiology. 2017 Oct;106(1):35–53. Available from: http://doi.wiley.com/10.1111/mmi.13752.

[35] Hoiczyk E. Structure and sequence analysis of Yersinia YadA and Moraxella UspAs reveal a novel class of adhesins. The EMBO Journal. 2000 Nov;19(22):5989–5999. Available from: http://emboj.embopress.org/cgi/doi/10.1093/emboj/19.22.5989.

[36] Fairman J, Dautin N, Wojtowicz D, Liu W, Noinaj N, Barnard T, et al. Crystal Structures of the Outer Membrane Domain of Intimin and Invasin from Enterohemorrhagic E. coli and Enteropathogenic Y. pseudotuberculosis. Structure. 2012 Jul;20(7):1233–1243. Available from: https://linkinghub.elsevier.com/retrieve/pii/S0969212612001700.

[37] Caveney NA, Caballero G, Voedts H, Niciforovic A, Worrall LJ, Vuckovic M, et al. Structural insight into YcbB-mediated beta-lactam resistance in Escherichia coli. Nature Communications. 2019 Dec;10(1):1849. Available from: http://www.nature.com/articles/s41467-019-09507-0.

[38] Oomen CJ, van Ulsen P, Van Gelder P, Feijen M, Tommassen J, Gros P. Structure of the translocator domain of a bacterial autotransporter. The EMBO Journal. 2004 Mar;23(6):1257–1266. Available from: http://emboj.embopress.org/cgi/doi/10.1038/sj.emboj.7600148.
